# Metric properties of the "timed get up and go- modified version" test, in risk assessment of falls in active women

**Published:** 2017-03-30

**Authors:** Margareth Lorena Alfonso Mora

**Affiliations:** 1 Programa de Fisioterapia, Universidad de La Sabana. Chia, Cundinamarca, Colombia

**Keywords:** Accidental falls, Adult, Aged, physical test, walking, cognition, task performance, psychomotor performance

## Abstract

**Objective:**

To analyse the metric properties of the Timed Get up and Go-Modified Version Test (TGUGM), in risk assessment of falls in a group of physically active women.

**Methods:**

A sample was constituted by 202 women over 55 years of age, were assessed through a crosssectional study. The TGUGM was applied to assess their fall risk. The test was analysed by comparison of the qualitative and quantitative information and by factor analysis. The development of a logistic regression model explained the risk of falls according to the test components.

**Results:**

The TGUGM was useful for assessing the risk of falls in the studied group. The test revealed two factors: the *Get Up* and the *Gait with dual task*. Less than twelve points in the evaluation or runtimes higher than 35 seconds was associated with high risk of falling. More than 35 seconds in the test indicated a risk fall probability greater than 0.50. Also, scores less than 12 points were associated with a delay of 7 seconds more in the execution of the test (*p*= 0.0016).

**Conclusions:**

Factor analysis of TGUGM revealed two dimensions that can be independent predictors of risk of falling: The Get up that explains between 64% and 87% of the risk of falling, and the Gait with dual task, that explains between 77% and 95% of risk of falling.

## Resumen

**Objetivo:**

Analizar las propiedades métricas del ***Test Timed Get Up and Go - modificado*** (TGUGM) midiendo el riesgo de caídas a un grupo de mujeres colombianas físicamente activas.

**Métodos:**

Estudio, transversal con 202 mujeres mayores de 55 años realizado en Chía, Colombia. Se aplicó el TGUGM para conocer su riesgo de caídas. La prueba comparó la información cualitativa y cuantitativa mediante un análisis factorial. El desarrollo de un modelo de regresión logístico explicó el riesgo de caídas según los componentes de la prueba.

**Resultados:**

El TGUGM permitió valorar el riesgo de caídas en el grupo estudiado. La prueba muestra dos factores: el Get up (*levantarse*) y el Gait with dual task (*Marchar con doble tarea*). Calificaciones inferiores a doce puntos y tiempos de ejecución superiores a 35 segundos reflejaron alto riesgo de caída. Un tiempo mayor a 35 segundos indica probabilidad de caída mayor a 0.50 y calificaciones menores a 12 puntos se asociaron con una demora de 7 segundos más en la ejecución del test (*p* <0.001).

**Conclusiones:**

El riesgo de caídas del grupo de mujeres colombianas fue alto en el 22%. El análisis factorial del TGUGM reflejó dos dimensiones que pueden ser predictoras independientes del riesgo de caída: El *Get up* que puede explicar entre el 64% y 87% del mismo y el *Gait with dual task*, que explica entre el 77% y el 95% de dicho riesgo.

## Introduction

Falls in older adults are a significant health problem. Approximately 30% of those older than 65 years fall at least once every year, and 6% of these falls result in fractures ^1^. Falls are considered a public health problem ^2^ because of the frequency of presentation, the associated morbimortality, and increased costs. They also constitute one of the best documented geriatric syndromes, are a marker of fragility ^3^, and are considered a death predictive factor by indirect causes ^4^. 

The risk of falls in the older adult is associated with a decrease in physical aptitude; the application of physical tests that measures falls to determine risk is therefore very relevant. Tinetti, Berg, and *Timed Get Up and Go* are the three most utilized tests*.* They evaluate a person's walking and balance aptitude, and the risk of falling is then determined based on this score ^5^. 

The Tinetti scale ^6^ has been a widely utilized measurement tool because prospective designs have concluded that it has a high predictive value for falls ^7^ ^,^ ^8^. However, the utilization of this test is limited due to its subjectivity and the moderately long period required to apply it.

The Berg scale provides information about the functional state of balance in older persons ^9^, and its validity, reliability, and sensitivity to change have been evidenced in other health conditions ^10^. For this test, the prediction of R^2^= 0.80 in healthy and active adults in community settings who were less than 70 years of age has been previously determined ^11^. In addition, a specificity of 93%-96% has been found for this scale ^12^. The sensitivity reported is lower but also acceptable, predicting fall risk at 53%-82.5%. However, because the Berg scale is a 14-item scale, it requires a large amount of time for its application.

The *Get Up and Go* test consists of performing the following actions: getting up from a chair, walking 3 m, passing around a cone, and returning to the chair as quickly as possible. This sequence is evaluated using a qualitative scale to measure performance: normal, very slightly abnormal, slightly abnormal, moderately abnormal, and extremely abnormal ^13^.

There was a first update of the *Get Up and Go* test in which time was included as the variable that determines the performance of the individual taking the test, as it is a variable that measures the individual's ability to carry out the instructions given. The finding from this update was that the longer the test was conducted, the lower the overall performance was, leading to a greater fall risk. This modification was called the *Timed Get Up and Go Test* (TGUG) ^14^. 

A more recent modification of the TGUG test, included other instructions in addition to walking. Specifically, the strength of the inferior limbs, coordination, balance, and walking, along with a cognitive task and other simultaneous motor tasks, are measured. The validation of this tool is pertinent because walking performance plus the addition of a simultaneous task imitate the motor activity processes that are found in adults' daily activities ^15^ ^-^ ^17^.

Therefore, the TGUGM test measures the time invested in completing the mentioned task and includes a qualitative assessment (QA) that allows possible zones of functional deficit to be isolated, helping health professionals to elaborate specific fall prevention strategies according to the subject's needs ^17^ ^.^

Considering that the TGUGM test provides information of motor skills in people, this study sought to analyse the psychometric properties of TGUGM test to measure the risk of falls in physically active Colombian women.

## Material and Methods

A cross-sectional study that validated the TGUGM was performed on 202 Colombian women from a group of 300 participants in municipality of Chia Colombia. The following inclusion criteria were applied: older than 55 to 75 years of age (age of retirement in the country), active participants in the Physical Activity Programme from the township of Chia. The exclusion criteria included: Women with cognitive deficiencies that prevent them from following simple orders, women who were not able to walk independently or who needed external help for walking, and women with the presence of acute mechanical lumbago, a vestibular or central nervous system disease diagnosis, or orthostatic hypotension. Both inclusion and exclusion criteria were identified and evaluated by an initial interview, which included sociodemographic and clinical information. 

This study was approved by Research Ethics Committee of the Universidad de La Sabana (Act No. 43 May 30, 2014), and all participants who completed the inclusion criteria provided informed consent for their inclusion in the study after being properly informed of the objective and the associated minimum risk according to the 2013 Declaration of Helsinki. 

In order to characterize the participants, their clinical history was used as source of the following data: age, educational level, marital status, occupation, history of falling within the last two years, number of medications, history of illness, and visual or auditory problems. These data were included in an analysis database. 

To evaluate the risk of falls, the TGUGM test was used. This is a physical test that measures balance and walking while performing a parallel cognitive task. The test is subdivided into six phases that are scored according to the time taken to complete each phase and the total time of the test. A QA for each phase using a 0 to 3 Likert scale was also conducted, with 0 being a deficient execution and 3 being an excellent execution.

**Figure 1:**
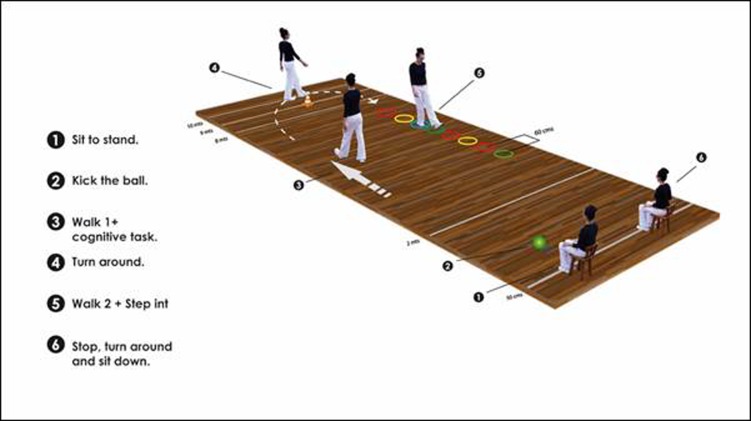
Timed Get Up and Go Test Modified Version (graphic description of test phases). Modifier from Giné Garriga 2009^16^

The first phase of the TGUGM is to stably get up from a 42-cm-tall chair without using one's hands. The second phase is to kick a 19 cm diameter ball weighing 0.2 kg without losing balance and with the greatest strength possible. The third phase is to walk toward a cone placed 10 m from the chair, while counting from 15 to 0 without changing walking speed and without making errors while counting. The fourth phase is passing around a cone, with the turn being stable enough to not touch the cone. Phase five corresponds to walking between rings placed at 60 cm, where participants are observed to see whether they step out of the rings. Finally, the sixth phase consists of the person sitting again in the chair with a controlled movement and without the help of their hands (Fig. 1).

After scoring each phase of the test, the scores are added together to obtain a total test result. Scores less than 12 indicate a high risk of falling ^17^.

The instructions given to each participant for performing the test were as follows: "Sit with your two hands resting on your lap. When you listen Get up and go, stand up without using your hands; kick the ball in front of you as strong as possible using your best foot, then walk at a comfortable pace while you count backwards from 15 to 0 around the cone without touching it, and return to the chair passing inside of the circles, trying not to touch any of their edges. Finally, sit down again, trying not to use your hands". One attempt to perform the test with each participant was made prior to the evaluation in order to verify that they understood all the instructions. 

To keep track of time, a Casio HS70W chronometer was used by the evaluator in each phase and activated when giving the order to start (counting from 1 to 3). The first time point (T1) corresponded to the time taken to sit down in the chair; the second time point (T2) was the time from standing to when the ball passed the 8 m line; the third time point (T3) was the time between kicking the ball and walking toward the cone, counting from 15 to 0; the fourth time point (T4)was when the person sat back down in the chair, and the chronometer was finally stopped to register the total time (TT) of the test. The performance of each phase of the test was evaluated in parallel using the QA and was conducted by a second evaluator who also calculated the total sum of the QA and gave a total score to all participants, indicating the importance of the result.

The results of the test were recorded in a matrix created with Microsoft Excel software, immediately after the test was performed. Later, with the assistance of an expert in Statistics, a factorial analysis was performed using test execution times according to the following steps: a correlation matrix between all times was constructed, including age and total QA score. Subsequently, the principal component analysis was used to select factors. To facilitate the interpretation of the result, the factors were rotated using the Varimax method. 

A dichotomous variable was created using the QA result that indicates the high or low level of risk of falling, keeping 12 points as a reference point. From this initial result, a comparison was made between groups using the Mann-Whitney U test. Finally, a binary logistic regression model was developed, and the execution times for the test were introduced to subtract the variable that predicts the risk level of falling according to the QA (greater or less than 12). A p-value < 0.05 was used for data analysis. 

## Results 

The mean age of participant women was 68 years (SD= 7.6). 34% had at least one fall within the previous two years. With the TGUGM, it was found that the average time to perform the test was 26 seconds and that the average result of the QA was 13 points (Table 1). 

**Table 1:** Description of characteristics of the sample

Variable		% Mean (n)
Age	55- 65	40.1 (81)
	66- 75	42.1 (85)
	>75	17.8 (36)
	Mean	68.4 ±7.6
Occupation (%)	Retired	30.0
	Employed	10.0
	Homemaker	60.0
	No schooling	13.4
Level of education (%)	Elementary	50.2
	Secondary school	21.9
	Technical	6.5
	University	5.0
	Post-graduate	3.0
History of falling in last two years (%)	Yes	34.2
Visual or auditory deficit	Visual	57.9
	Auditory	23.8
	Both	3.5
Total time TGUGM	Mean	26.8 ± 6.2
Calificación EC TGUGM	Mean	13.11 ± 2.2

TGUGM: Timed get up and Go modified test

A factorial analysis of the matrix data was made, searching for correlations. Findings showed that correlations were statistically different from 0, except for T2 and T3 (Table 2). Prior to the factorial analysis, the Bartlett statistical test was performed, yielding a *p* value less than 0.001, reaffirming the correlation between the variables. 

**Table 2:** Correlation matrix between times of the test TGUGM, QA and age

	Age	Total score	TT	T1	T2	T3
Age						
Total score QA	-0.194^**^					
Total time TGUGM	0.324^**^	-0.448^**^				
T1	0.189^**^	-0.166^*^	0.292^**^			
T2	0.170^**^	-0.342^**^	0.397^**^	0.170^**^		
T3	0.241^**^	-0.304^**^	0.803^**^	0.156^**^	0.014	
T4	0.257^**^	-0.394^**^	0.881^**^	0.230^**^	0.417^**^	0.652^**^

TGUGM: Timed get up and Go modified test

QA: qualitative assessment TGUGM

T: time. Therefore, T1= time one; T2= time two; T3= time three; T4= time four; and TT= total time.

*p >0.05 **p >0.01

Utilising the principal component analysis and the previously mentioned Varimax rotation method, the factor analysis finally produced the rotated component matrix (Table 3) in which two statistical factors were visualised, the first of them associated with T3 and T4 and the second with T1 and T2. The first factor explains walking with the cognitive and balance task *(Gait with dual task*, (GDT), and the second factor explains the phases for incorporation and strength of the inferior limbs after kicking the ball (*Get up*, (GU)). 

**Table 3:** Rotated components factorial analysis matrix

	Factor 1 Gait with dual task (Walk with cognitive and motor dual task)	Factor 2 Get Up (Get Up and Kick the Ball)
T1	0.195	0.645
T2	-0.029	0.874
T3	0.951	-0.061
T4	0.771	0.479

T: time. Therefore, T1= time one; T2= time two; T3= time three; T4= time four; and TT= total time.

From the studied 22% of women had a high risk of falls with scores under 12 points according to the QA. The other 78% of the participants had a low risk of falls. Later, the differences in test execution times were calculated (Table 4). All times were significantly different according to the results from the Mann-Whitney U test, with the greatest times in subjects who scored under 12 (with a high risk of falling). The difference in TT was 7 seconds on average, with the time being greater for persons with a high risk of falling according to the QA. 

**Table 4:** Mean difference in times according to the risk of falling*

	With risk (QA >12) n= 45		Without risk (QA <12) n= 157	
	Mean	95% CI	Mean	95% CI
TT	32.40	30.40-34.30	25.30	24.40-26.10
T1	0.94	0.83-1.05	0.77	0.73-0.81
T2	4.03	3.49-4.56	2.80	2.56-3.03
T3	12.50	11.40-13.60	9.80	9.30-10.30
T4	14.30	13.10-15.40	11.60	11.20-12.04

QA: Qualitative assessment

* All *p* value <0.00.1Mann-Whitney U

T: time. Therefore, T1= time one; T2= time two; T3= time three; T4= time four; and TT= total time.

When conducting the logistic regression, the dependent variable was considered the risk of falling according to the QA. From there, the models were adjusted, including times and finally choosing the total time to explain the risk. The mathematical model showed that a time greater than 35 seconds indicated a probability greater than 0.50 for a participant to obtain also a QA score of less than 12 points (β Value of Total time TGUGM 0.29 *p*= 0.001; β Value Constant -7.252 *p*= 0.001). 

**Figure 2:**
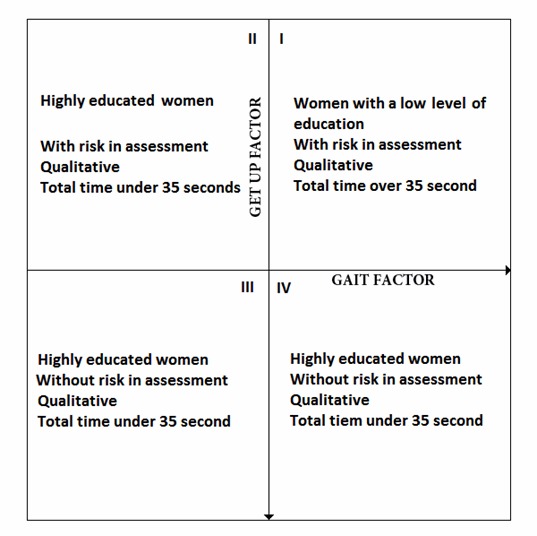
Distribution of the factors according to the sample condition (age an educative level)

The results of the factorial analysis were reconsidered when the factors were located on the Cartesian coordinate plane (X= *Gait with dual task*; Y= *Get up*). This exercise showed that quadrants II, III, and IV mainly incorporated women with university educational level and higher, with a low fall's risk according to the QA, and a total test execution time of less than 35 seconds. However, quadrant I differs from the characteristics of the three other quadrants, with women with lower educational levels scoring less than 12 on the QA and exhibiting a test execution time of greater than 35 seconds (Fig. 2). 

## Discussion 

In describing the level of risk of falls of 202 active, cognitively intact and independent Colombian women, it was found that 22% of them have a high risk of falls. These findings corroborate previous international reports that showed high level of risk of falls in older people ^2^. 

The evidenced properties of the TGUGM when tested with physically active Colombian women, showed two dimensions: the first, related to physical qualities for GU and the second associated with GDT. According to Giné-Garriga ^15^, the physical qualities related to GU (times T1 and T2 according to the factorial analysis) are inferior limb strength. This finding was corroborated using the concurrent validity tests for inferior limb strength, finding a relationship with the *five chair stand* (0.69) test and with the maximum isometric contraction of the knee extensors (0.69). The GDT phase (times T3 and T4) are related to coordination, balance, and walking. In addition, the concurrent validity study of the TGUGM test showed that this factor was associated with the *Gait speed* test (0.77-0.84) ^15^. 

When comparing the predictive capacity of *Timed Get Up and Go* with *Gait speed* tests, it was concluded that both tests predict functional decline, difficulty in daily life activities, and falls ^18^. These tests are highly related to each other, although the TGUGM can measure more physical qualities simultaneously, providing more information about an older adult's physical condition.

According to the TGUGM validity construction ^15^, GDT is associated with the risk of falling, coinciding with what was affirmed by Muhaidat *et al*. ^19^, who performed a 6-month follow-up of a group of older adults after having administered simple, double, and triple motor tests. This study revealed that the GDT test best predicted falls, at least in the study population (n = 66). 

The factor grouping GDT could explain the relative complexity of the motor sequence implied in the development of the TGUGM. Consequently, the highest levels of education yielded results that are not associated with the level of falls risk according to the QA. In the same quadrant of the factorial analysis, women with QA scores higher than 12 and with TT less than 35 seconds showed through logistical regression that a TT greater than 35 seconds indicated a probability higher than 0.50 of having a score lower than 12 in the QA, which in this instance agrees with the findings from Giné-Garriga ^17^. When conducting the TGUGM test validity construct, these investigators divided a sample of older adults according to their physical activity level and according to reports of falls within the previous 6 months. They found that sedentary adults with a history of falls within the previous 6 months had an average TT of 36.8 seconds, which agrees with the results of this report. 

Time is one of the determinants of the variations in the *Timed Get Up and Go* test because, according to the systematic literature review conducted by Shoene *et al*. ^20^, any variation in execution times is significantly different, with adults who present falls having the greatest execution times. Therefore, establishing a cut-off of 35 seconds for determining the risk level of falls according to the TGUGM may serve as a reference point for future prospective designs. 

## Conclusions 

The TGUGM properties were divided into 2 dimensions: GDT and GU*,* which were grouped into quadrant 1 of the factorial analysis, having a QA score lower than 12. The time that best explains the high risk of falling is a total time greater than 36 seconds. In addition, it is clear that the test time scores were significantly different according to the qualitative evaluation of the TGUGM, with the highest times being among women with scores less than 12 points. 

The TGUGM test is an easy to apply physical tool that may be used in different scenarios where there is a risk of falls. It is necessary to establish prospective research designs in order to complement the TGUGM study in its accuracy for falls prediction. 
